# High-Quality *de novo* Chromosome-Level Genome Assembly of a Single *Bombyx mori* With *BmNPV* Resistance by a Combination of PacBio Long-Read Sequencing, Illumina Short-Read Sequencing, and Hi-C Sequencing

**DOI:** 10.3389/fgene.2021.718266

**Published:** 2021-09-16

**Authors:** Min Tang, Suqun He, Xun Gong, Peng Lü, Rehab H. Taha, Keping Chen

**Affiliations:** ^1^School of Life Sciences, Jiangsu University, Zhenjiang, China; ^2^Institute of Clinical Pharmacology, Anhui Medical University, Hefei, China; ^3^Department of Medical Rheumatology, Columbia University, New York, NY, United States; ^4^Department of Sericulture, Plant Protection Research Institute, Agricultural Research Center, Giza, Egypt

**Keywords:** *Bombyx mori*, PacBio sequencing, genome assembly, illumine sequencing, Hi-C technology

## Abstract

The reference genomes of *Bombyx mori* (*B. mori*), Silkworm Knowledge-based database (SilkDB) and SilkBase, have served as the gold standard for nearly two decades. Their use has fundamentally shaped model organisms and accelerated relevant studies on lepidoptera. However, the current reference genomes of *B. mori* do not accurately represent the full set of genes for any single strain. As new genome-wide sequencing technologies have emerged and the cost of high-throughput sequencing technology has fallen, it is now possible for standard laboratories to perform full-genome assembly for specific strains. Here we present a high-quality *de novo* chromosome-level genome assembly of a single *B. mori* with nuclear polyhedrosis virus (*BmNPV*) resistance through the integration of PacBio long-read sequencing, Illumina short-read sequencing, and Hi-C sequencing. In addition, regular bioinformatics analyses, such as gene family, phylogenetic, and divergence analyses, were performed. The sample was from our unique *B. mori* species (NB), which has strong inborn resistance to *BmNPV*. Our genome assembly showed good collinearity with SilkDB and SilkBase and particular regions. To the best of our knowledge, this is the first genome assembly with *BmNPV* resistance, which should be a more accurate insect model for resistance studies.

## Introduction

*Bombyx mori* (*B. mori*) or domestic silkworm is a well-known bioreactor that produces natural silk in sericulture and is frequently used as a lepidopteran model to study insect immunology and disease resistance (Maekawa et al., 1988; Arunkumar et al., 2006). In recent decades, the genetics and genomics of *B. mori* in several Asian countries have been greatly elucidated (Xia et al., 2004). Its natural primal enemy, *B. mori* nucleopolyhedrovirus (*BmNPV*), can cause widespread death to the overwhelming majority of *B. mori* strains. Several strains bred in different laboratories display congenital *BmNPV* resistance (Xu et al., 2013). Similar to most higher organisms, the key prerequisite of most biological research is the assembly of whole-genome sequences. For *B. mori*, the first genome sequence with 3× coverage generated by shotgun sequencing technology (fosmid-end) was published in Japan in 2004, and the *B. mori* strain used was *p50T* (Koike et al., 2003; Mita et al., 2004). At almost the same time, an independent Chinese group also performed a similar whole-genome sequencing (BAC-end) with 5.9× coverage, and the *B. mori* strain used was *Dazao* (Xia et al., 2004). Both studies declared that the estimated genome coverage was more than 90%. Based on the EST database in the former project, the first version of SilkBase was released in 2006. Then, the integrated transcriptomic and genomic data were added to the project and released in the second version of SilkBase in 2015. Two years later, the *p50T* strain was sequenced again by making use of the combination of PacBio and Illumina sequencing technologies, and this genome assembly with increased accuracy was released as SilkBase v2.1 (Kawamoto et al., 2019). Furthermore, the Silkworm Knowledge-based database (SilkDB) was developed for data storage, retrieval, analysis, and visualization by Chinese groups in 2005 (Wang et al., 2005), and the assembly accuracy was later promoted by the International Silkworm Genome Consortium in 2010 (SilkDB 2.0) (International Silkworm Genome, 2008). Ten years later, cutting-edge PacBio and Hi-C biotechnologies were employed to assemble the latest version of the *Dazao* genome in 2020 (SilkDB 3.0) (Duan et al., 2010; Lu et al., 2020). To the best of our knowledge, SilkDB 3.0 and SilkBase v2.1 are the most commonly used database reference genomes. Hence, they are recognized as the basis for a number of *B. mori* studies and databases (Cao et al., 2017; Li et al., 2019; Zhu et al., 2019; Fujimoto et al., 2020).

It is a pity that both of the strains they used do not have *BmNPV*-resistance. Perhaps, as a consequence, their genome information is not perfectly accurate for the resistance studies considering the importance of the reference genome. Since countless efforts have been made for the *BmNPV* resistance without any acknowledged results, we think that it is time to optimize the reference genome group—for example, establish a *BmNPV*-resistant genome. In addition, in our opinion, the pipelines for constructing the above-mentioned two genome references could be more accurate and practical. For SilkDB 3.0, short-read sequencing data that could correct the draft genome assembly were not added to aid the assembly process (Jayakumar and Sakakibara, 2019). For SilkBase, Hi-C data should be used to help arrange and orient contigs or scaffolds (Belton et al., 2012). More importantly, neither *Dazao* nor *p50T* has resistance to *BmNPV*, which may increase genomic noise or even introduce errors in the resistance study of *B. mori*. Hence, here we introduce a high-quality *de novo* chromosome-level genome assembly of *B. mori* with *BmNPV* resistance by integrating data from PacBio long-read sequencing, Illumina short-read sequencing, and Hi-C sequencing technology.

## Materials and Methods

### Sample Collection and DNA Isolation

Two five-instar *B. mori* belonging to the *NB* strain were collected from the School of Life Sciences, Jiangsu University, and downstream wet-laboratory experiments were performed at Novogene Co., Ltd. First, one of the imagoes was sufficiently ground with polyvinyl polypyrrolidone powder, and whole DNA was extracted for sequencing using sodium dodecyl sulfate (SDS) and cetyltrimethylammonium bromide (Chen et al., 2010; Kasajima, 2018). Then, 1% agarose gel was used to examine DNA degradation and contamination. Consequently, the DNA purity was checked using a NanoPhotometer^®^ spectrophotometer (IMPLEN, CA, United States), and the DNA concentration was measured using a Qubit^®^ DNA Assay Kit on a Qubit^®^ 2.0 fluorometer (Life Technologies, CA, United States).

### Library Construction and Illumina Sequencing

A total of 1.5 μg extracted DNA was used as input material for the DNA sample preparations and sheared with a Covaris Focused ultrasonicator. Following the recommendations of the manufacturer, a library for Next-Gen sequencing was constructed using a Truseq^®^ Nano DNA Sample Preparation Kit (Illumina, San Diego, CA, United States) after end-repair and the addition of index code. Subsequently, the fragments were end-polished, A-tailed, and ligated with the full-length adapter for Illumina sequencing with further PCR amplification. After fragment selection for a size of 350 bp and PCR amplification, the products were purified with the AMPure XP system (PacBio, Menlo Park, CA, United States), and the library was analyzed for size distribution by an Agilent 2100 Bioanalyzer and quantified using real-time PCR. Finally, the library constructed as detailed above was sequenced by an Illumina HiSeq X Ten instrument, and 150 bp paired-end (PE) reads, including ∼350 bp inserts, were generated. After removing the reads with adapters, the paired reads that had more than 10% N bases or over 20% low-quality bases on either end sequence were discarded. As a result, the remaining reads passed onto the downstream analysis as clean reads.

### Library Construction and PacBio Sequencing

More than 5 μg of concentrated genomic DNA (gDNA) that passed the inspections was used for the size-select DNA fragment step. g-TUBE was utilized to shear the gDNA into ∼20-kb fragments to construct a 20-kb SMRTbell library. After shearing, AMPure PB beads were used to concentrate the sheared gDNA. Then, the fragments were treated to remove the single-strand overhangs and repair DNA damage with ExoVII enzyme. Subsequently, the ends of the double-strand fragments were polished with T4 DNA Polymerase and then ligated to single-molecule real-time (SMRT) hairpin adapters. Through EXOIII and VII enzyme treatment, the SMRTbell templates were washed out. The library was again concentrated and purified with AMPure PB beads. In this case, we employed the BluePippin system (Sage Science, Inc.) and set a 20-kb cutoff threshold for size selection. The sublibrary was also concentrated and purified with AMPure PB beads. After annealing the sequencing primer to the SMRTbell template, polymerase was bound to both ends of the SMRTbell templates using a binding kit for efficient loading into ZWMs. Finally, a total of 14 SMRT cells were run on the PacBio Sequel system with the P6-C4 sequencing reagent (Kingan et al., 2019). Briefly, a SMRTbell library was constructed with the SMRTbell Express Template Prep Kit 2.0 according to the latest protocol published by Pacific Biosciences of California, Inc.

### Hi-C Experiment and Sequencing

The Hi-C experiment was performed as described previously (Belton et al., 2012; Yardimci et al., 2019). Cell suspensions were made with the other *B. mori* imago and treated with paraformaldehyde to fix the three-dimensional structures of the nuclei to retain the relationship between genomic and physical distance. Therefore, after cell lysis with RIPA lysis buffer (1 M Tris–HCl, pH 8, 1 M NaCl, 10% CA-630, and 13 units of protease inhibitor), the exposed chromatin was cross-linked *in situ* to trap sequence interactions across the entire genome and between different chromosomes. Then, the supernatant was centrifuged at 5,000 rpm at room temperature for 10 min. The obtained pellet was washed twice with 100 μl ice cold 1× NEB buffer, followed by centrifugation at 5,000 rpm for 6 min.

The chromatin was resuspended in 100 μl NEB buffer and solubilized with dilute SDS, followed by incubation at 65°C for 10 min. After quenching SDS with Triton X-100, overnight digestion was performed with the four-cutter endonuclease *Mbo*I at 37°C on a rocking platform. Subsequently, the fragmented chromatin was digested with the restriction enzymes *Hind*III and *Dpn*II at 37°C for 16 h. Next, for proximity ligation, the fragmented loci were ligated and marked with biotin to create chimeric junctions between adjacent sequences after incubation at 37°C for 45 min. The enzymatic system was inactivated with 20% SDS solution. Finally, proteinase K was added for reverse cross-linking overnight at 65°C, and the resulting sample was purified and dissolved in 90 μl of double-distilled water. The purified fragments were sheared to a size of ∼350 bp, and then their ends were repaired. The biotin-containing fragments were isolated with Dynabeads^TM^ M-280 Streptavidin purchased from Thermo Fisher Scientific Inc. After adding A-tails to the fragment ends and following ligation by Illumina PE sequencing adapters, the Hi-C library was amplified by 12–14 PCR cycles and sequenced on the Illumina NovaSeq 6000 platform with the PE set to 150 bp.

### RNAseq Experiment and Transcriptome Sequencing

Transcriptome sequencing was performed as described by Conesa et al. (2016). The RNA pool was made from a whole one-instar *B. mori* using the RNeasy Maxi Kit purchased from QIAGEN, and raw sequencing data were obtained on the Illumina HiSeq X sequence platform. The subsequent quality control steps were performed with FastQC, FASTX-Toolkit, and Trimmomatic (Bolger et al., 2014).

## Results

### *De novo* Assembly of the *B. mori* Genome and Assessment

The raw Illumina sequencing data first underwent quality control after image recognition, contamination, and adapter elimination. Then, the initial characterization of the *B. mori* genome was estimated through *k*-mer (*k* = 17 in this study) analysis of the clean data by jellyfish and GenomeScope (Vurture et al., 2017). As a result, the estimated genome size (475.39 and 464.90 Mbp after later correction), heterozygosity (0.23%), and repeat content (43.78.09%) were basically consistent with previous findings (Mita et al., 2004; Xia et al., 2004; International Silkworm Genome, 2008; Kawamoto et al., 2019). All the sequencing data produced in this study are listed in Table 1. As the table shows, long-read sequencing on the PacBio Sequel system yielded 50.08 G of data with a high coverage of 107.72× short-read sequencing on the Illumina NovaSeq 6000 system yielded 64.81 G of data with an average coverage of 139.41× and Hi-C sequencing on the HiSeq X system yielded 83.78 G of data with an average coverage of 180.21×.

**TABLE 1:** Statistics of the *Bombyx mori* sequencing data for genome assembly.

Types	Approach	Sequencing platform	Insert size (bp)	Total data (G)	Read length (bp)	Sequence coverage (×)^*a*^
Genome	Illumina	Novaseq 6000	350	64.81	150	139.41
Genome	Pacbio	PacBio Sequel	–	50.08	∼20k	107.72
Genome	Hi-C	HiSeq X	–	83.78	150	180.21
Transcriptome Total	Illumina	HiSeq X	350	198.67	–	427.34

*^a^The coverage was calculated according to an estimated genome size of 464.90 Mbp.*

During the assembly process, the long reads downloaded from the PacBio Sequel platform were subjected to self-correction by the NextCorrect module of NextDenovo.^1^ Taking the corrected reads as input materials, FALCON was employed to assemble the draft framework of the *B. mori* genome (Chin et al., 2016). To correct the high error rate of the long-read sequencing technology (Rang et al., 2018), two genome sequence polishing steps were performed: the Quiver algorithm was first used to polish the genome using PacBio long reads (Chin et al., 2013), and another round of genome-wide base-level correction of the Illumina clean reads was performed using Pilon (Walker et al., 2014). For chromosome-level scaffolding (Oluwadare et al., 2019), the clean Hi-C reads were mapped to the assembled genome using BWA (Li and Durbin, 2010), and the repeats and unmapped reads were removed by SAMtools (Li et al., 2009) to obtain high-quality reads and information about the restriction sites for enzyme cutting. As a result, only uniquely mapped read pairs were considered for subsequent analysis, and the Hi-C heatmap did not show misassembly during scaffolding (Supplementary Figure 1). LACHESIS was finally used to cluster, order, and orient the assembled contigs (Burton et al., 2013). The assembly statistics of the completed genome are shown in Table 2 and Supplementary Tables 1–3. Only scaffolds larger than 100 bp were selected to perform the assembly; the contig N50 was 3.75 Mbp, and the scaffold N50 was 17.26 Mbp.

**TABLE 2:** Statistics of the *B. mori* genome assembly.

Sample ID	Length			Number
	Contig^*a*^ (bp)	Scaffold (bp)	Contig^*a*^	Scaffold
				
Total	455,458,164	455,459,137	241	70
Max	12,311,683	21,717,039	–	–
Number ≥2,000	–	–	241	70
N50	3,751,516	17,263,692	42	12
N60	2,941,979	16,129,549	57	15
N70	2,487,328	15,544,771	73	18
N80	1,798,075	14,680,113	95	21
N90	1,077,501	12,534,506	126	24

*^*a*^Contig after scaffolding.*

**FIGURE 1:**
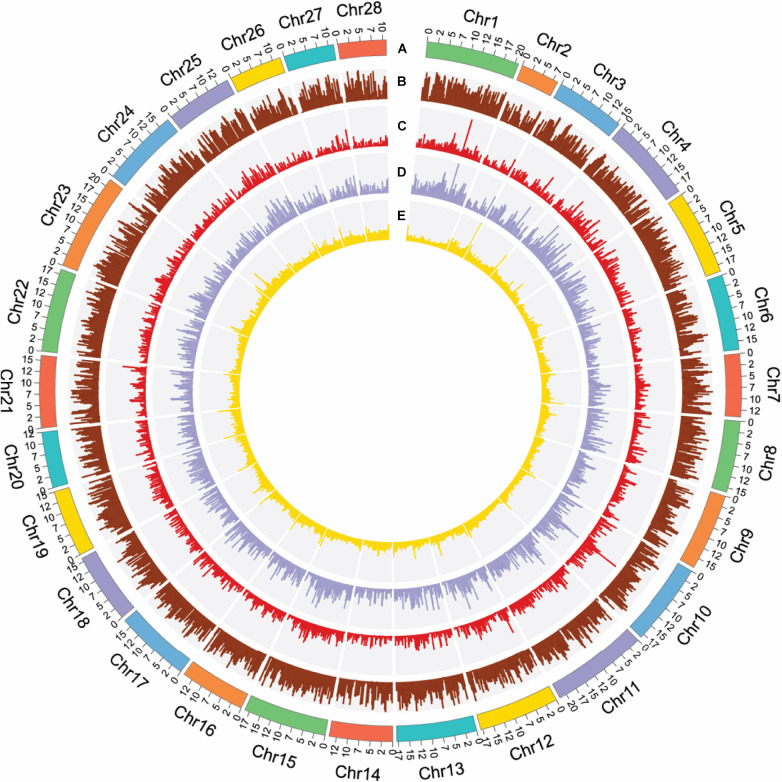
Summary of gene distribution and genetic diversity across the 28 chromosomes. **(A)** Chromosome position. **(B)** Gene density in 200-kb windows. **(C)** CDS density in 200-kb windows. **(D)** Exon density in 200-kb windows. **(E)** GC content in 200-kb windows.

Briefly, we assembled a high-quality chromosome-scale genome for *B. mori de novo* by making use of the 198.67 G of sequencing data (Figure 1). The overall length and N50 value of the contigs were 455.46 and 3.75 Mbp, respectively, while the overall length and N50 value of the scaffolds were 455.46 and 17.26 Mbp. To evaluate the quality of the newly assembled genome, its completeness was first assessed by the core eukaryotic genes mapping approach (CEGMA), whose core gene database includes 248 genes derived from six model eukaryotes (Parra et al., 2007). Supplementary Table 4 shows that 223 core eukaryotic genes were retrieved, which was up to 89.92% of the total number of genes. In addition to CEGMA, Benchmarking Universal Single-Copy Ortholog (BUSCO) software, invoking TBLASTN (Gertz et al., 2006), AUGUSTUS (Stanke and Morgenstern, 2005), and HMMER (Wheeler and Eddy, 2013), was employed (Seppey et al., 2019). Of the 978 orthologous genes, 98.1% complete BUSCOs (C + D) were detected in its report (see Supplementary Tables 4, 5). CEGMA and BUSCO revealed that we obtained a high-quality and complete *B. mori* genome.

**FIGURE 2:**
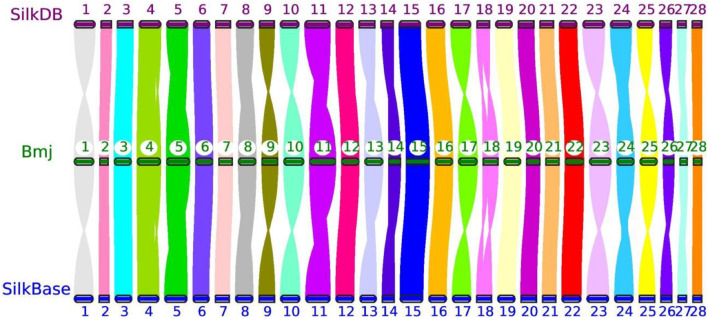
Genomic collinearity of three chromosome-level genomes—*Bmj*, Silkworm Knowledge-based database (SilkDB), and SilkBase—by JCVI. The number in the circle represents the chromosome identity for each strain.

Moreover, the reference genomes from SilkDB and SilkBase were used to check the collinearity with our genome assembly with JCVI (Tang et al., 2008). As expected, great collinearity existed among the three chromosome-level genomes (Figure 2). The distortions on several chromosomes, such as Chr1, Chr10, and Chr23, were attributable to scaffold perversions, which is quite reasonable, as large chromosomal structure changes may occur among closely related strains. In addition, optical mapping methods and approaches for the ordering and orientation of scaffolds can also cause a systematic deviation (Ekblom and Wolf, 2014; Belser et al., 2018; Deschamps et al., 2018). Nevertheless, crevices with different sizes indicate small INDELs that may be the origins of BmNPV resistance; this will be analyzed in detail in future studies in our laboratory.

### Genome Annotation

Supplementary Figure 2 shows three routes in the road map of the genome annotation phase. From the left, an integration of homology-based and *de novo* approaches was used to identify the repeat sequences. First, LTR_FINDER^2^ (Xu and Wang, 2007), RepeatScout,^3^ and RepeatModeler^4^ were employed to create a *de novo* repeat sequence dataset. Then, based on the RepBase database, RepeatMasker (see text footnote 3) and RepeatProteinMask^5^ were used to recognize the sequences that had a high similarity in RepBase. Moreover, Tandem Repeats Finder (Benson, 1999) and RepeatModeler2 (Flynn et al., 2020) were also utilized to detect tandem repeats and transposable element (TE) families in the assembled genome with default settings. Consequently, all the above-mentioned repetitive elements were combined to annotate the genome assembly using RepeatMasker (Table 3 and Supplementary Figure 3; Supplementary Table 6).

**TABLE 3:** Statistics for the classification of repetitive elements.

	*De novo* + Repbase length (bp)	% in genome	TE protein length (bp)	% in genome	Combined TE length (bp)	% in genome
DNA	43,928,578	9.64	8,097,056	1.78	48,033,677	10.55
LINE	151,419,612	33.25	40,869,238	8.97	162,254,924	35.62
SINE	36,753,778	8.07	0	0	36,753,778	8.07
LTR	46,826,667	10.28	8,338,880	1.83	47,903,023	10.52
Other	0	0	0	0	0	0
Unknown	2,722,614	0.6	0	0	2,722,614	0.6
Total	253,233,852	55.6	57,220,911	12.56	257,512,897	56.54

*De novo + Repbase is a transposable element (TE) pool generated by the integration of RepBase and the combination of RepeatModeler, RepeatScout, and LTR_FINDER based on the 80-80-80 principle of Uclust. TE proteins are also a TE pool derived from the application of RepeatProteinMask to the RepBase protein database. Combined TEs are the integration of De novo + Repbase and TE proteins after removing overlaps. Unknown refers to repetitive elements that cannot be classified by RepeatMasker.*

For the prediction of gene structures, *de novo*, homology-based, and transcriptome-based strategies were integrated to predict genes in the *B. mori* genome (Table 4 and Supplementary Figure 2). *De novo* prediction was performed with AUGUSTUS (Stanke and Morgenstern, 2005), GlimmerHMM (Majoros et al., 2004), SNAP (Johnson et al., 2008), Geneid,^6^ and Genscan (Burge and Karlin, 1997), while homology-based prediction was conducted with BLAT, which aligns the protein sequences of *Ame*, *Pra*, *Bmo*, *Pxy*, *Tca*, and *Dme* to the above-mentioned genome assembly (Kent, 2002). Then, the transcript reads were aligned to the genome assembly using the TopHat package (Kim et al., 2013), followed by gene structure prediction with Cufflinks (Ghosh and Chan, 2016). Finally, all gene models were merged by EVidenceModeler (EVM)^7^ (Haas et al., 2008) after removing redundancy. To enrich the contents, the RNAseq sequence data were assembled *de novo* with Trinity (Grabherr et al., 2011; Haas et al., 2013) and applied to insert untranslated regions and alternative splicing information to the EVM dataset by PASA (Haas et al., 2003). In conclusion, 9,113 gene structures were synchronously supported by *de novo*, homology-based, and transcriptome-based prediction strategies (Supplementary Figure 5). The same prediction pipeline was also applied to several proximal species (*Bmj*, *Ame*, *Bmo*, *Dme*, *Pra*, *Pxy*, and *Tca*; see Supplementary Table 10). Notably, the number of predicted genes for *Bmo was* 13,850, which was very close to the 13,103 predicted genes for *Bmj*.

**TABLE 4:** Statistics of gene structure annotation.

	Gene set	Number	Average transcript length (bp)	Average CDS length (bp)	Average exons per gene	Average exon length (bp)	Average intron length (bp)
*De novo*	Augustus	11,666	9,995.37	1,376.92	6.04	227.91	1,709.45
	GlimmerHMM	48,042	8,378.78	538.88	3.37	160.04	3,312.04
	SNAP	22,743	11,183.17	645.71	4.06	158.86	3,438.46
	Geneid	9,281	33,434.53	779.78	6.32	123.39	6,138.85
	Genscan	13,002	23,083.74	1,343.72	6.02	223.36	4,334.14
Homolog	*Ame*	9,438	6,629.92	1,047.42	4.46	234.63	1,611.49
	*Bmo*	32,454	4,125.35	908.63	3.04	298.64	1,574.88
	*Dme*	6,852	7,308.34	1,179.68	4.99	236.56	1,537.21
	*Pra*	27,430	4,470.74	1,002.10	3.23	310.71	1,558.78
	*Pxy*	20,272	4,816.18	1,392.41	3.28	424.03	1,499.16
	*Tca*	20,637	3,647.42	1,130.13	2.7	418.63	1,481.13
RNAseq	PASA	36,779	16,791.09	1,516.73	7.27	208.72	2,437.34
	Cufflinks	35,742	26,014.39	4,582.43	8.24	556.26	2,961.05
	EVM	14,449	12,572.50	1,297.05	5.9	219.83	2,301.05
	Pasa-update^*a*^	13,773	15,181.10	1,452.26	6.59	220.32	2,455.20
	Final set^*b*^	13,103	14,748.03	1,444.25	6.53	221.28	2,407.13

*^*a*^Includes UTRs.*

*^*b*^Includes UTRs; generated from the longest transcript after alternative splicing and redundant single-exon elimination by PASA2 update.*

For gene function annotation, the final protein sequences obtained above were used as inputs for the SwissProt,^8^ Nr,^9^ Kyoto Encyclopedia of Genes and Genomes (KEGG),^10^ InterPro,^11^ Gene Ontology,^12^ and Pfam^13^ databases. After alignment to these published protein databases, 99.6% of the genes were annotated with functional entities (Supplementary Table 8). In addition, non-coding RNAs were also annotated (Supplementary Table 9). tRNAs were detected with tRNAscan-SE^14^ (Lowe and Chan, 2016; Chan and Lowe, 2019), while miRNAs and snRNAs were detected with the INFERNAL module of Rfam^15^ (Kalvari et al., 2018, 2021).

### Genomic Comparison

Genomic comparison in bioinformatics analysis, including gene family clustering and phylogenetic and divergence analysis, was performed by comparing our in-house *B. mori* genome assembly with the assemblies of a set of 17 other representative species. In addition to three closely related species (*Bmo*, *Aya*, and *Bma*) and five other lepidopteran species (*Sfr*, *Pxy*, *Pra*, *Har*, and *Dpl*), three dipteran species (*Aya*, *Aae*, and *Dme*), three hymenopteran species (*Ame*, *Lhu*, and *Nvi*), and two coleopteran species (*Lde* and *Tca*) were also included in the comparison list. Moreover, *Caenorhabditis elegans*, belonging to Rhabditia, was also included. For convenience, their abbreviations are summarized in Supplementary Table 10.

**FIGURE 3:**
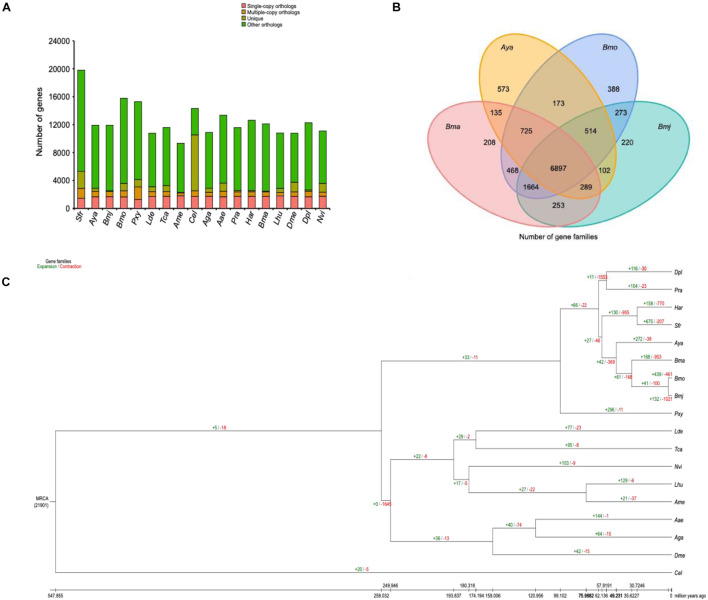
Gene family and phylogenetic and divergence analyses of *Bombyx mori* and other representative insect genomes. **(A)** Clusters of orthologous and paralogous gene families in *B. mori* and 17 other sequenced insect genomes. The *y*-axis indicates the number of gene families, while the *x*-axis indicates the 18 species. The total number of gene families was 21,951, of which the number of single-copy orthologs was 620. **(B)** Venn diagram representing the distribution of shared gene families among *B. mori* and three other silkworms (*Bma*, *Aya*, *Bmo*, and *Bmj*). **(C)** Gene family expansions and contractions estimated by CAFÉ v3.0 and CAFE_fig. The green and red numbers indicate expanded and contracted gene families, respectively.

First, OrthoMCL^16^ was used for gene family clustering analysis (Li et al., 2003; Chen et al., 2006) using the following parameters: mode, 3; inflation, 1.5 blast_file, gg_file. As a result, a total of 21,904 gene families were identified, and 650 strict single-copy orthologs were recovered in the 18 genomes (Figure 3A). In addition, a core set of 6,897 gene families was shared by *Bmj* (for *shipshape*, use *Bmj* instead of *B. mori*), *Bmo*, *Bma*, and *Aya* (Figure 3B), and only 220 gene families uniquely belonged to *Bmj.* A consequent large-scale analysis found 84 gene families specific to *Bmj* but not the 17 other selected species. Most of these specific genes were involved in receptor activity, transmembrane signaling receptor activity, and glycosphingolipid biosynthesis (Supplementary Figure 7).

Second, CAFÉ v3.0^17^ and CAFE_fig^18^ were used to analyze changes in the gene family size using the following parameters (-i -t 8 -l -p 0.05). As Figure 3C shows, 132 gene families were expanded, while 1,021 gene families were contracted from the *Bmj* genome. In comparison with the closely interlinked *Bmo*, which gained 439 gene families and missed 461 gene families, *Bmj* lost many more gene families. These missed genes were rich in diverse functions, such as catalytic activity, fatty acid biosynthesis, fatty acid metabolism, and AMPK signaling pathway (Supplementary Figure 8). Corresponding to the phylogenetic analysis performed with the mcmctree program of the PAML package^19^ (parameters: rootage, 1,000; clock, 3; alpha, 0.603820) (Yang, 1997), Figure 3A shows that *Bmj* has a very close relationship to *Bmo* and phylogenetically diverged from the common ancestor ∼3 million years ago. However, the history of human breeding is up to 5,000 years long (Tabunoki et al., 2016; Meng et al., 2017), and the best explanation is manual intervention in sericulture, which increases their divergence. By using the phylogenetic analysis as a supplement, the above-mentioned 650 single-copy gene families were subjected to internal alignment with MUSCLE,^20^ and then the aligned results were merged to form a super alignment matrix (Edgar, 2004a, b). After that, the ML TREE algorithm in RAxML^21^ was used with default parameters to create a phylogenetic tree (Figure 3C; Stamatakis, 2014).

**FIGURE 4:**
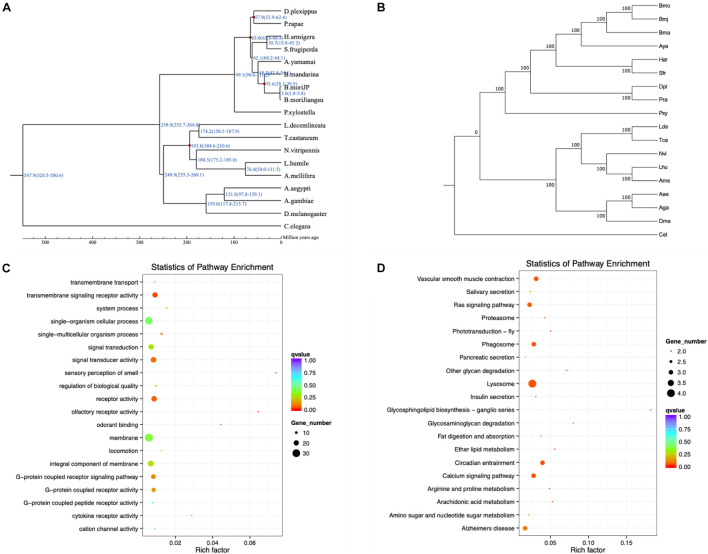
Evolutionary analysis of *B. mori* by Gene Ontology (GO) and Kyoto Encyclopedia of Genes and Genomes (KEGG) pathway enrichment analysis. **(A)** Divergence time estimation of *B. mori* and closely related species. The divergence time is shown at the branches of the phylogenetic tree, along with the confidence intervals in parentheses. **(B)** Phylogenetic tree of *Bmj* species. **(C,D)** GO and KEGG pathway enrichment analysis of positively selected genes related to *BmNPV* resistance.

To detect positively selected genes related to different phenotypes of *Bmj*, a positive selection analysis was executed in three diverse combinations with the other 17 species. MUSLCE was again employed to align the protein sequences of the single-copy gene families between foreground branches and background branches. Then, the aligned results were cleaned by removing the low-quality regions using Gblocks and then reversed to CDS^22^ (Castresana, 2000). For each gene family, the branch-site model in the codeml program of the PAML package was applied to check whether it was positively selected in the *Bmj* branch (Yang, 1997). Instead of simply seeking genes with ka/ks > 1, PAML makes use of two hypothesized likelihood ratios to double-check the positive selection (Yang and Nielsen, 2002; Zhang et al., 2005) (Figures 4A,B). For the eating pattern of mulberry (Ge et al., 2018a, b), the foreground branches were *Bmj*, *Bmo*, and *Bma*, and the background branches were *Aya*, *Har*, *Sfr*, *Pra*, *Dpl*, and *Pxy* in the first group. Sixty-two targeted genes that were involved in metabolic processes, catalytic activity, and Huntington’s disease were obtained (Supplementary Figure 9). For the capability of silk production, the foreground branches were *Bmj*, *Bmo*, *Bma*, and *Aya*, and the background branches were *Lhu*, *Ame*, *Nvi*, *Lde*, *Tca*, *Aae*, *Aga*, and *Dme* in the second group. A total of 119 targeted genes were enriched in metal ion binding, zinc ion binding, and transmembrane transport (Supplementary Figure 10). In the third group, for resistance to *BmNPV*, the foreground branch was *Bmj*, while the background branches were *Bmo*, *Har*, *Sfr*, *Pra*, *Dpl*, *Pxy*, *Bma*, and *Aya*. The downstream analysis indicated that the *BmNPV* resistance of our NB species may be related to pathways of transmembrane signaling receptor activity and lysosome and signal transducer activity (Figures 4C,D and Supplementary Figure 11).

## Conclusion and Discussion

Since approximately 5,000 years ago, *B. mori* has been one of the most important agricultural economic insects for silk production in Asia (Xiang et al., 2018). As it is completely domesticated in sericulture, its survival and reproduction totally depend on human beings (Jiang and Xia, 2014). With advances of biotechnology, *B. mori* has been treated as an important bioreactor for producing recombinant protein (Tomita et al., 2000, 2003). Moreover, because of their intermediate genome size, short life cycle, affordability, and ability to be easily used for drug screening, *B. mori* is a perfect model organism for scientific discovery, especially for lepidopterans, and is useful for economic research (Kaito et al., 2002; International Silkworm Genome, 2008; Tabunoki et al., 2016; Meng et al., 2017). To maintain the consistency of biological background among different laboratories, the *B. mori* strains of *Dazao* and *p50T* were first separately sequenced and annotated in China and Japan by whole-genome shotgun sequencing in 2004, and the genome sequence has been updated every few years, which is considered the gold standard (Mita et al., 2004; Xia et al., 2004; Meng et al., 2017; Kawamoto et al., 2019).

According to a search of NCBI PubMed with the keywords “(*Bombyx mori*) OR (silkworm),” 7,024 relevant works have been published since 2004. Among these, transcriptomics studies have inevitably made use of the above-mentioned reference genomes to identify transcripts and quantify gene expression. To obtain the brain transcriptome profiles of *BmNPV*-infected and non-infected silkworm larvae, Wang et al. (2015) mapped their clean reads to the SilkDB reference genome. Moreover, Li et al. (2016) comprehensively investigated the transcriptomic changes between susceptible and resistant *B. mori* strains after *BmNPV* infection. Three years later, they (Li et al., 2019) updated the analysis of the different alternative splicing events based on the same reference genome. Recently, Sun Q. et al. (2020) also downloaded genome sequences and annotation files from the SilkDB website for the transcriptome analysis of the immune response of *B. mori* after bidensovirus infection at the early stage. Based on the SilkBase genome sequence, Shoji et al. (2013) characterized a novel chromodomain-containing gene and later (Shoji et al., 2014) identified a number of genes whose expression can be enhanced by heterochromatin protein 1. Therefore, it follows that the two reference genomes with non-resistance to *BmNPV* are being widely used as the *B. mori* “Bible.” However, with the increasing demand for precise reference genomes, a personalized edition is needed for *B. mori* research. With our more refined reference genome, one can find precise bioinformatics information such as SNPs and INDELs related to *BmNPV* resistance.

Thanks to the rapid advances in sequencing technology and bioinformatic tools in the past decade, transcriptome sequencing (∼$130/sample) has become a cost-effective technology for obtaining biological information. However, the emerging third-generation sequencing technologies, such as Nanopore/PacBio long-read sequencing and Hi-C sequencing, require a larger budget than that of standard laboratories (Makałowski and Shabardina, 2019; Wang et al., 2019). Even the use of whole-genome sequencing on the popular Illumina platform to generate short reads for draft assembly correction is expensive, let alone optical genome mapping (i.e., Bionano maps), which can correct chimeric contigs and scaffolding errors caused by Hi-C sequencing (Chan et al., 2018; Bocklandt et al., 2019; Choi et al., 2020; Ning et al., 2020; Sun L. et al., 2020). In addition, the entire pipeline of genome assembly is a tedious and time-consuming process, as ideal computing modules should be constructed for each individual species. As such, methods and algorithms have been developed or improved by scientists with different professional backgrounds (Shin et al., 2019; Nakabayashi and Morishita, 2020). Thus, there is a need for many users to find a single universal pipeline for datasets from different species for more accurate results (Minervini et al., 2020). In short, for the further analysis of species, an updated reference genome with a high resolution is needed to enhance the robustness of the final results.

## Data Availability Statement

The final chromosome assembly was submitted to NCBI and deposited in the BioProject under accession number PRJNA721561.

## Author Contributions

KC designed the study. MT and SH performed the data analysis and wrote the manuscript. PL and XG revised the manuscript. All the authors made a direct and intellectual contribution to this topic and approved the article for publication.

## Conflict of Interest

The authors declare that the research was conducted in the absence of any commercial or financial relationships that could be construed as a potential conflict of interest.

## Publisher’s Note

All claims expressed in this article are solely those of the authors and do not necessarily represent those of their affiliated organizations, or those of the publisher, the editors and the reviewers. Any product that may be evaluated in this article, or claim that may be made by its manufacturer, is not guaranteed or endorsed by the publisher.

## Footnotes

1
https://github.com/Nextomics/NextDenovo

2
http://tlife.fudan.edu.cn/tlife/ltr_finder

3
http://www.repeatmasker.org

4
http://www.repeatmasker.org/RepeatModeler.html

5
https://github.com/rmhubley/RepeatMasker/blob/master/RepeatProteinMask

6
https://genome.crg.es/geneid.html

7
http://evidencemodeler.sourceforge.net/

8
https://www.uniprot.org/

9
https://www.ncbi.nlm.nih.gov/refseq/about/non-redundantproteins/

10
https://www.genome.jp/kegg/

11
https://www.ebi.ac.uk/interpro/

12
http://geneontology.org/docs/go-annotations/

13
http://pfam.xfam.org/

14
http://lowelab.ucsc.edu/tRNAscan-SE/

15
http://infernal.janelia.org/

16
http://orthomcl.org/orthomcl/

17
https://hahnlab.github.io/CAFE/index.html

18
https://github.com/LKremer/CAFE_fig

19
http://abacus.gene.ucl.ac.uk/software/paml.html

20
http://www.drive5.com/muscle/

21
http://sco.h-its.org/exelixis/web/software/raxml/index.html

22
http://molevol.cmima.csic.es/castresana/Gblocks_server.html
