# Supplementary material for: High-Quality de novo Chromosome-Level Genome Assembly of a Single Bombyx mori With BmNPV Resistance by a Combination of PacBio Long-Read Sequencing, Illumina Short-Read Sequencing, and Hi-C Sequencing
Source: Front Genet. 2021 Sep 16;12:718266. doi: 10.3389/fgene.2021.718266 (PMC8481875; doi:10.3389/fgene.2021.718266)
Supplement: Supplementary file 1 [file Data_Sheet_1.docx]

*Supplemental File*

**High-quality *de novo* chromosome-level genome assembly of a single** ***Bombyx mori* with** ***BmNPV* resistance** **by a combination of PacBio long-read sequencing, Illumina short-read sequencing and Hi-C sequencing**

Min Tang^1^, Suqun He^1^, Xun Gong^2,3^, Peng Lü^1^, Rehab H. Taha^4^, Keping Chen^1,^*

1. *School of Life Sciences, Jiangsu University, Zhenjiang, Jiangsu, China*
2. *Institute of Clinical pharmacology,* *Anhui Medical University, Hefei, Anhui, China*
3. *Department of Medical Rheumatology, Columbia University, New York, NY, USA*
4. *Department of Sericulture, Plant Protection Research Institute, Agricultural Research Center,* *Cairo, Egypt*

*** Corresponding author: Keping Chen ([kpchen@ujs.edu.cn](mailto:kpchen@ujs.edu.cn))

Number of Pages: 17

Number of Figures:11

Number of Tables: 10

**SUPPLEMTNAL TABLES**

Table S1 | Statistics of nucleotide composition for the *B. mori* genome assembly

|  | Number (bp) | % of genome |
| --- | --- | --- |
| **A** | 140,506,149 | 30.85 |
| **T** | 140,443,676 | 30.84 |
| **C** | 87,254,636 | 19.16 |
| **G** | 87,253,703 | 19.16 |
| **N** | 973 | 0 |
| **Total** | 455,459,137 | - |
| **GC** | 174,502,346 | 38.31 |

* GC content of the genome without N

Table S2 | Statistics of read coverage for the *B. mori* genome assembly

|  |  | Percentage |
| --- | --- | --- |
| **Reads** | Mapping rate (%) | 98.92 |
|  | Average sequencing depth | 122.99 |
| **Genome** | Coverage (%) | 99.82 |
|  | Coverage at least 4X (%) | 99.73 |
|  | Coverage at least 10X (%) | 99.62 |
|  | Coverage_at_least_20X（%） | 99.4 |

Table S3 | Statistics of SNPs for the *B. mori* genome assembly

|  | Number | Percentage (%) |
| --- | --- | --- |
| **All SNPs** | 239,098 | 0.0529 |
| **Heterozygosis SNPs** | 238,703 | 0.0528 |
| **Homology SNPs** | 395 | 0.0001 |

Table S4 | CEGMA assessment for the *B. mori* genome assembly

| species | complete | | complete + partial | |
| --- | --- | --- | --- | --- |
|  | # Prots | %completeness | # Prots | %completeness |
| *B.mori* | 214 | 86.29 | 223 | 89.92 |

Table S5 | BUSCO assessment for the *B. mori* genome assembly

| Species | BUSCO notation assessment results |
| --- | --- |
| B. mori | C:98.1%[S:97.4%,D:0.7%],F:0.6%,M:1.3%,n:978 |

Table S6 | Statistics of repetitive elements detected by an integration of homology-based and de novo approaches

| Type | Repeat Size(bp) | % of genome |
| --- | --- | --- |
| TRF | 7,523,991 | 1.65 |
| RepeatMasker | 253,233,852 | 55.6 |
| ProteinMask | 57,220,911 | 12.56 |
| Total | 260,014,481 | 57.09 |

Table S7 | Statistics of gene structures from proximal species

| **Species** | **Number** | **Average transcript length(bp)** | **Average CDS length(bp)** | **Average exons per gene** | **Average exon length(bp)** | **Average intron length(bp)** |
| --- | --- | --- | --- | --- | --- | --- |
| Bmj | 13,103 | 14,748.03 | 1,444.25 | 6.53 | 221.28 | 2,407.13 |
| Ame | 9,922 | 12,155.20 | 1,824.06 | 7.09 | 257.38 | 1,697.23 |
| Bmo | 13,850 | 14,511.88 | 1,448.47 | 6.9 | 209.88 | 2,213.65 |
| Dme | 13,918 | 4,492.75 | 1,610.48 | 3.96 | 406.49 | 973.11 |
| Pra | 12,471 | 9,576.77 | 1,600.94 | 7.58 | 211.24 | 1,212.33 |
| Pxy | 18,106 | 9,648.64 | 1,406.53 | 7.07 | 198.91 | 1,357.58 |
| Tca | 12,863 | 6,802.14 | 1,565.09 | 5.3 | 295.39 | 1,218.38 |

The gene prediction of several proximal species were also annotated using the same pipeline for *Bmj*. Note that *Bmo* have the close number of genes.

Table S8 | Statistics of gene function annotation

|  | **Number** | **Percent (%)** |
| --- | --- | --- |
| Total | 13,103 | - |
| SwissProt | 9,736 | 74.3 |
| Nr | 12,619 | 96.3 |
| KEGG | 9,452 | 72.1 |
| InterPro | 12,969 | 99 |
| GO | 11,959 | 91.3 |
| Pfam | 9,395 | 71.7 |
| Annotated | 13,051 | 99.6 |
| Unannotated | 52 | 0.4 |

Table S9 | Statistics of annotation for noncoding RNA

|  | **Type** | **Copy(w)^†^** | **Average length(bp)** | **Total length(bp)** | **% of genome** |
| --- | --- | --- | --- | --- | --- |
| miRNA |  | 11,744 | 96.82 | 1,137,101 | 0.25 |
| tRNA |  | 513 | 74.83 | 38,387 | 0.008428 |
| rRNA | rRNA | 373 | 96.4 | 35,959 | 0.007895 |
|  | 18S | 9 | 189 | 1,701 | 0.000373 |
|  | 28S | 2 | 109 | 218 | 0.000048 |
|  | 5.8S | 4 | 115.25 | 461 | 0.000101 |
|  | 5S | 358 | 93.8 | 33,579 | 0.007373 |
| snRNA | snRNA | 601 | 139.28 | 83,708 | 0.018379 |
|  | CD-box | 12 | 132.25 | 1,587 | 0.000348 |
|  | HACA-box | 9 | 181.44 | 1,633 | 0.000359 |
|  | splicing | 577 | 138.35 | 79,829 | 0.017527 |

† genome-wide annotation.

Table S10 | Abstract of 18 species compared in this study

| Chinese Name | Name | Abbr. |  | Gene | Order |
| --- | --- | --- | --- | --- | --- |
| 草地夜蛾 | Spodoptera frugiperda | Sfr |  | 22086 | lepidoptera |
| 日本天蚕 | Antheraea yamamai | Aya |  | 13610 | lepidoptera |
| 江苏家蚕 | Bombyx mori Jiangsu | Bmj |  | 13053 | lepidoptera |
| 日本家蚕 | Bombyx_mori_JP | Bmo |  | 16815 | lepidoptera |
| 小菜蛾 | Plutella xylostella | Pxy |  | 17441 | lepidoptera |
| 马铃薯甲虫 | Leptinotarsa Decemlineata | Lde |  | 12671 | loleoptera |
| 赤拟谷盗 | Tribolium castaneum | Tca |  | 12786 | loleoptera |
| 蜜蜂 | Apis mellifera | Ame |  | 9881 | hymenoptera |
| 秀丽隐杆线虫 | Caenorhabditis elegans | Cel |  | 20060 | rhabditia |
| 冈比亚按蚊 | Anopheles gambiae | Aga |  | 12311 | diptera |
| 埃及伊蚊 | Aedes aegypti | Aae |  | 14535 | diptera |
| 菜青虫 | Pieris rapae | Pra |  | 12064 | lepidoptera |
| 棉铃虫 | Helicoverpa armigera | Har |  | 13258 | lepidoptera |
| 中国野桑蚕 | Bombyx mandarina | Bma |  | 12520 | lepidoptera |
| 阿根廷蚁 | Linepithema humile | Lhu |  | 11428 | hymenoptera |
| 黑腹果蝇 | Drosophila melanogaster | Dme |  | 13554 | diptera |
| 大红斑蝶 | Danaus plexippus | Dpl |  | 14514 | lepidoptera |
| 蝇蛹金小蜂 | Nasonia vitripennis | Nvi |  | 12834 | hymenoptera |

**SUPPLEMTNAL FIGURES**

**
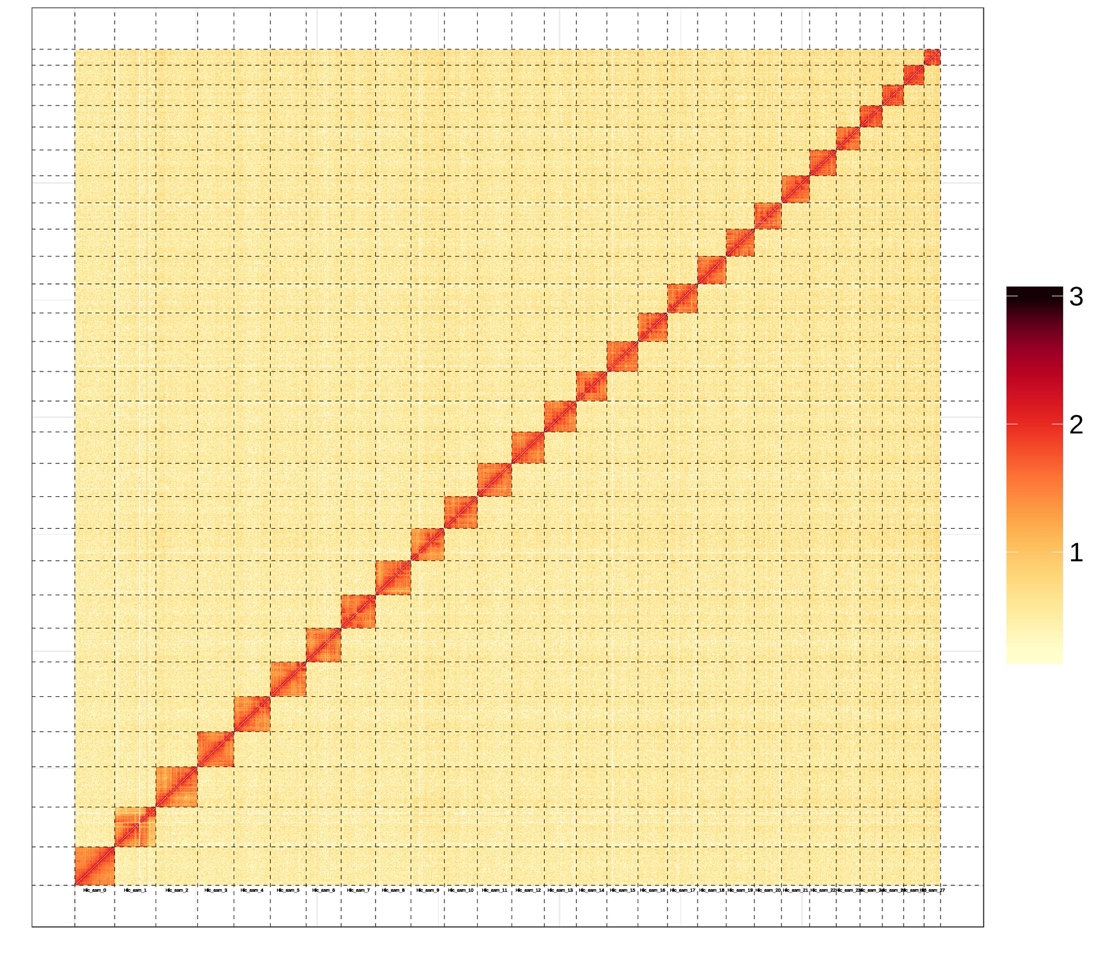
**

Figure S1 | **Hi-C heatmap after correcting the assembled genome.** Up to 99.54% Hi-C data were mapped the genome polished by Illumina short-read sequence data. The 28 blocks on the diagonal line indicates the 28 chromosome-level scaffolds.

**
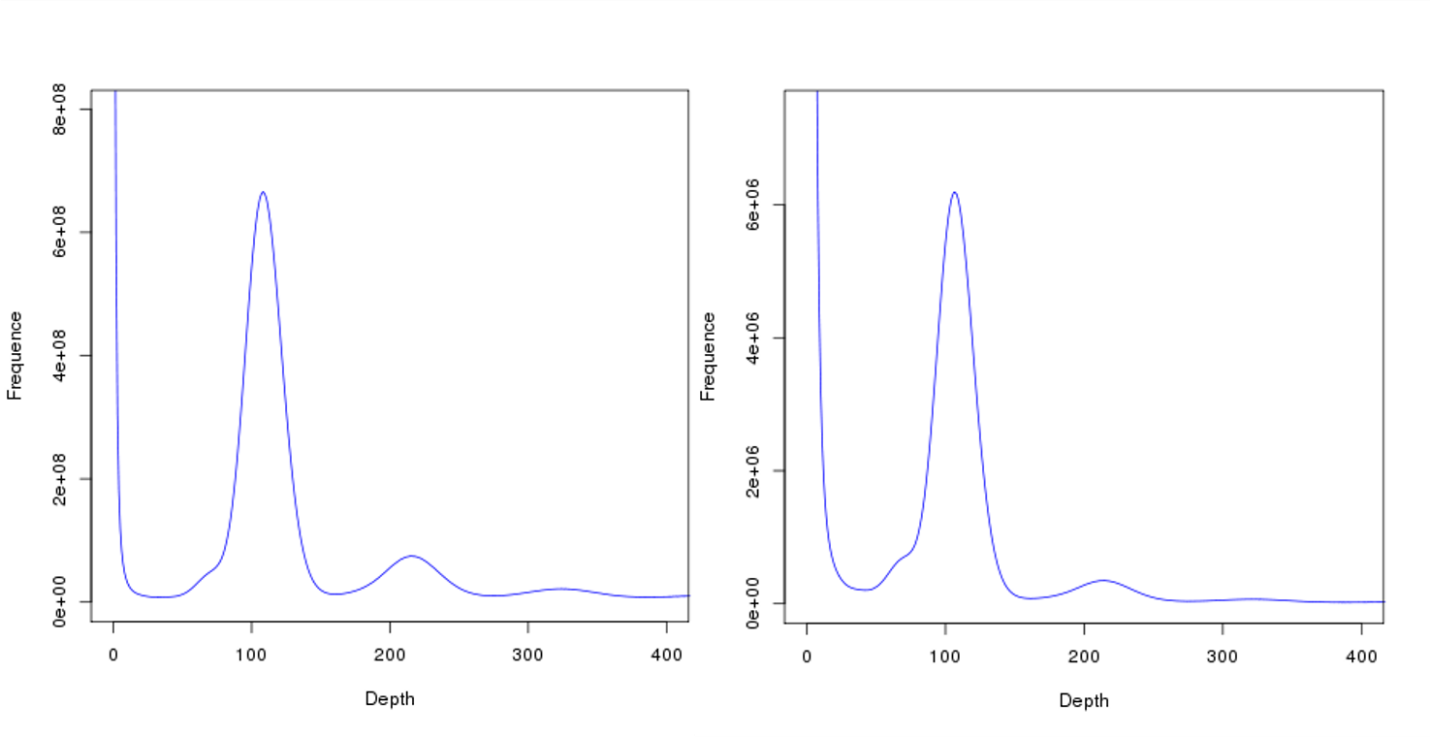
**

Figure S2 | **Characters of *B.mori* genome generated by Illumina short-read analysis**. (Left) The peak of the 17 kmer analysis is around 107. Based on the formula of Kmer-number/depth, the estimated genome size was 475.39Mbp. After revision, the final genome size was 494.90Mbp with 0.23% heterozygosity and 43.78% repeat content. (Right) Distribution of depth and the number of kmer variety.


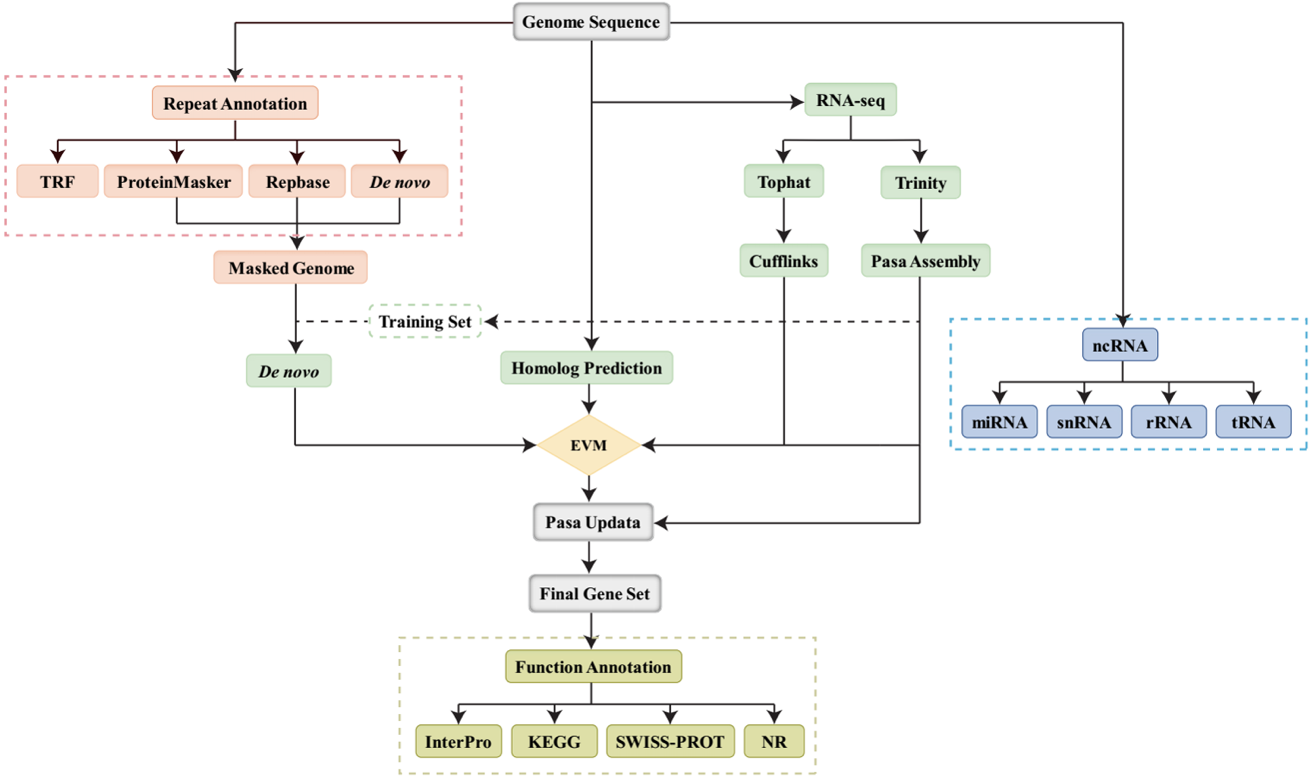


Figure S3 | **Workflow of genome annotation**.


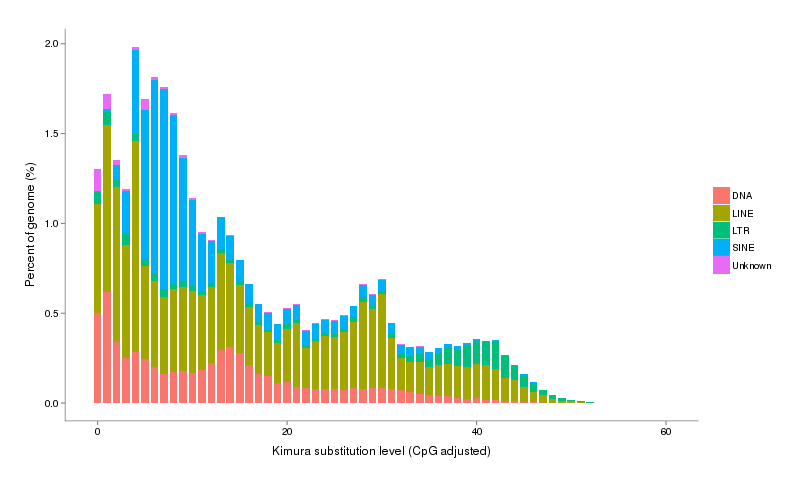


Figure S4 | **Distribution of TE sequence divergence derived from the application of RepeatMasker on Repbase.**


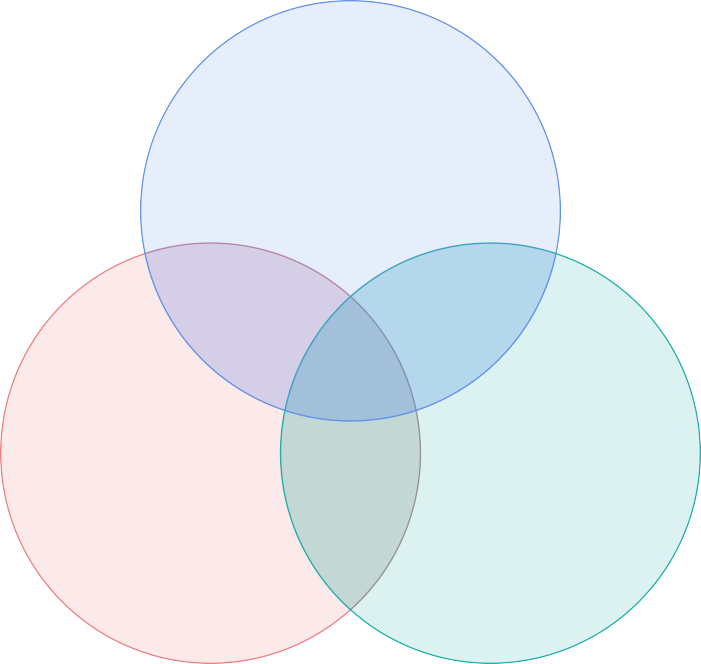


denovo_0.5

78

920

904

9113

9

28

102

homolog_0.5

rna_0.5

Figure S5 | **Evidence support of gene structure prediction**. 9113 gene structures were synchronously supported by *De novo*, homology-based and transcriptome-based prediction strategies

Figure S6 | **Graphical representation for gene structures from proximal species**.


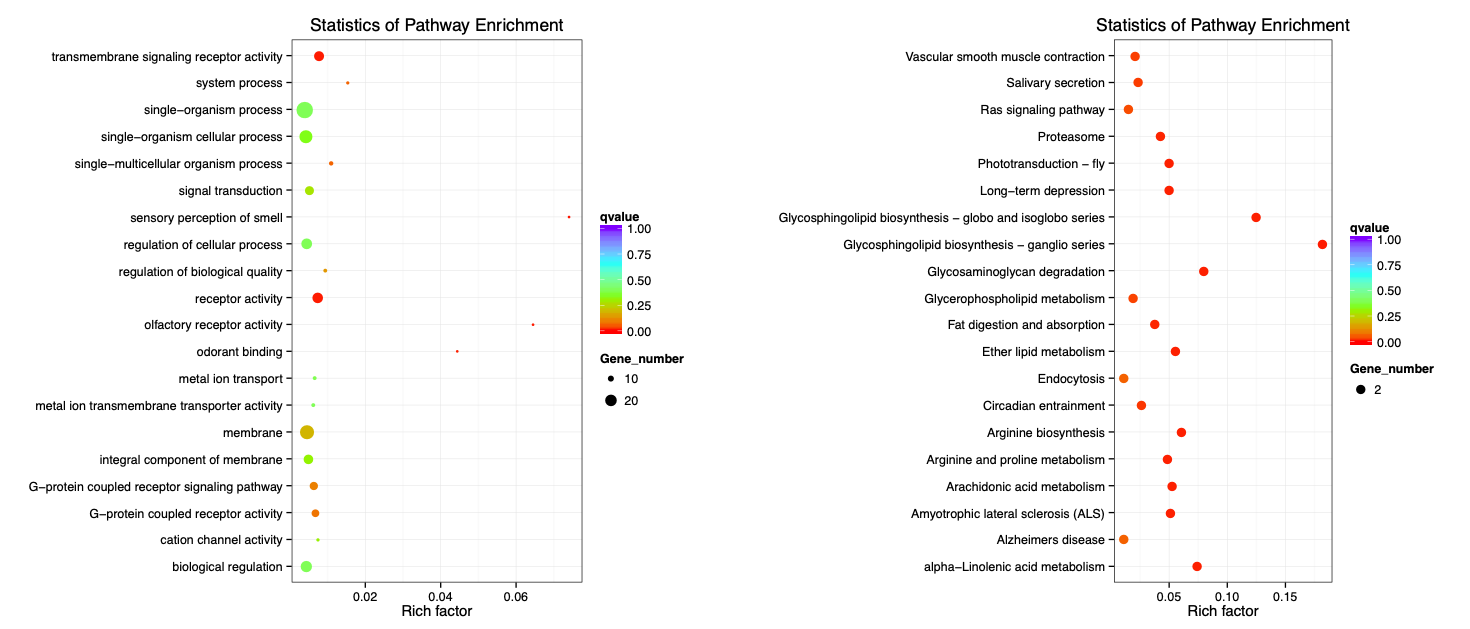


Figure S7 | **Pathway enrichment of unique gene faminlies in *Bmj***. (Left) Results from GO database; (Right) Results from KEGG database.


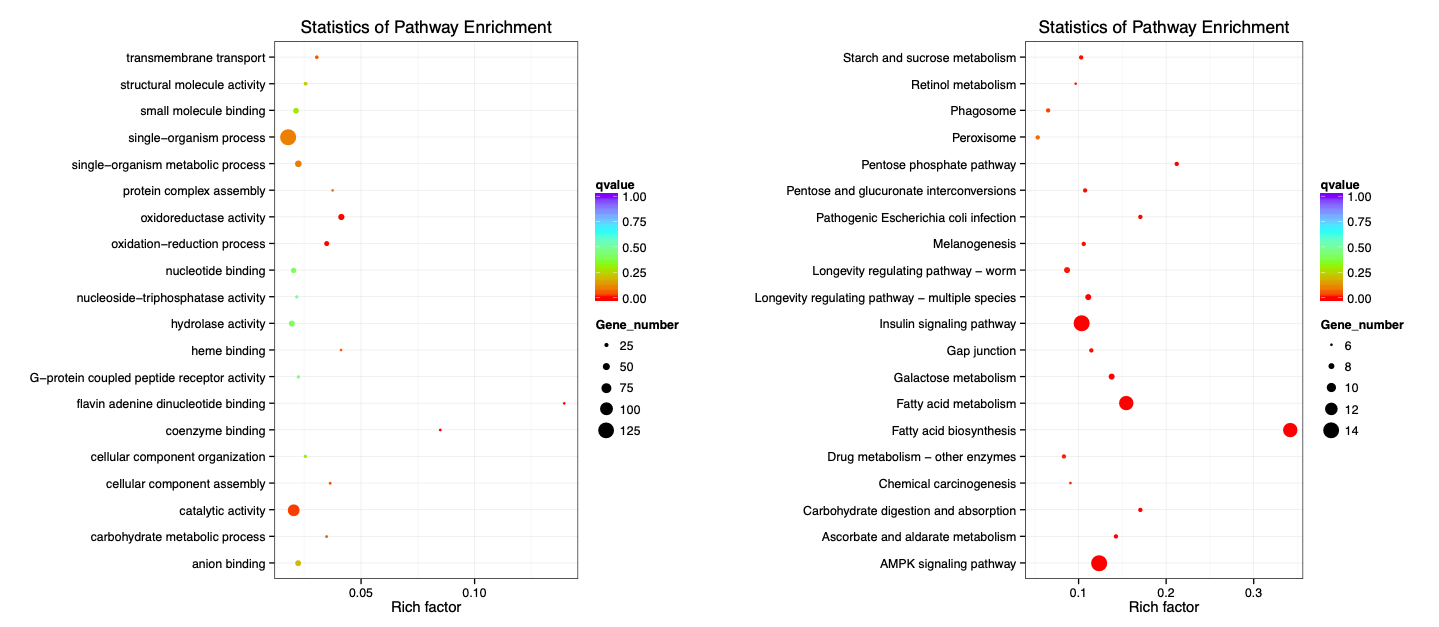


Figure S8 | **Pathway enrichment of contracted gene faminlies in *Bmj***. (Left) Results from GO database; (Right) Results from KEGG database.]


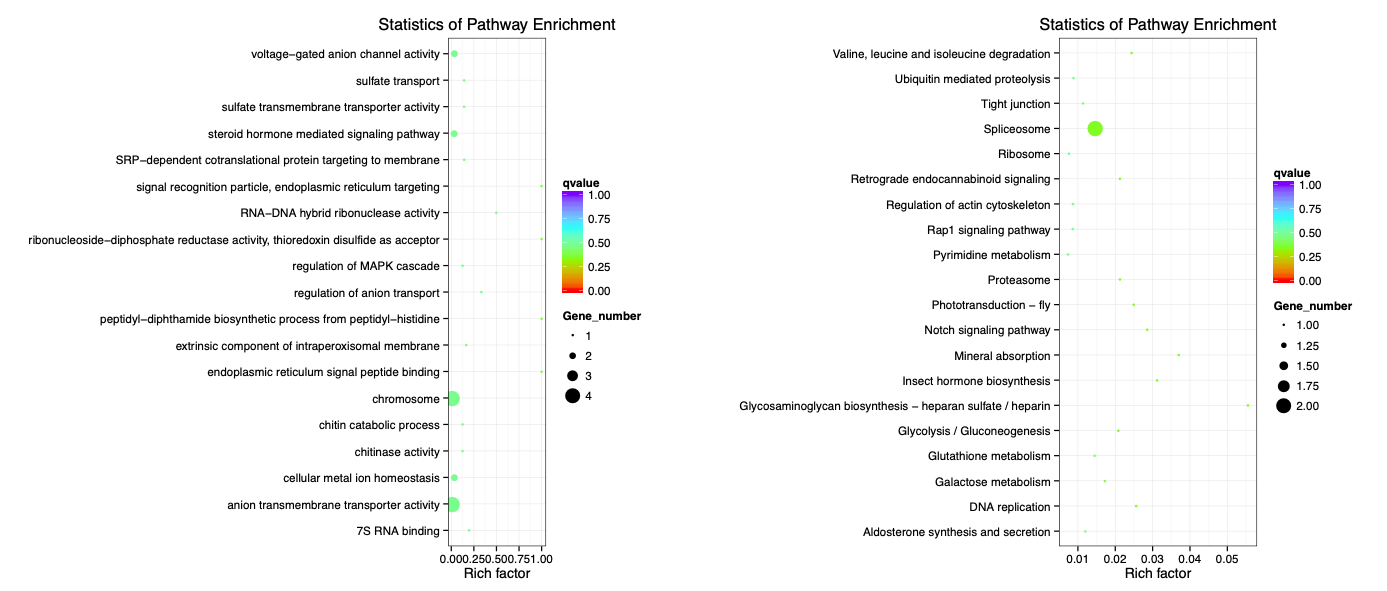


Figure S9 | **Pathway enrichment of positively selected genes for the eating pattern of mulberry**. The foreground branches were Bmj, Bmo and Bma and the background branches were *Aya*, *Har*, *Sfr*, *Pra*, *Dpl* and *Pxy* in the first group . (Left) Results from GO database; (Right) Results from KEGG database.


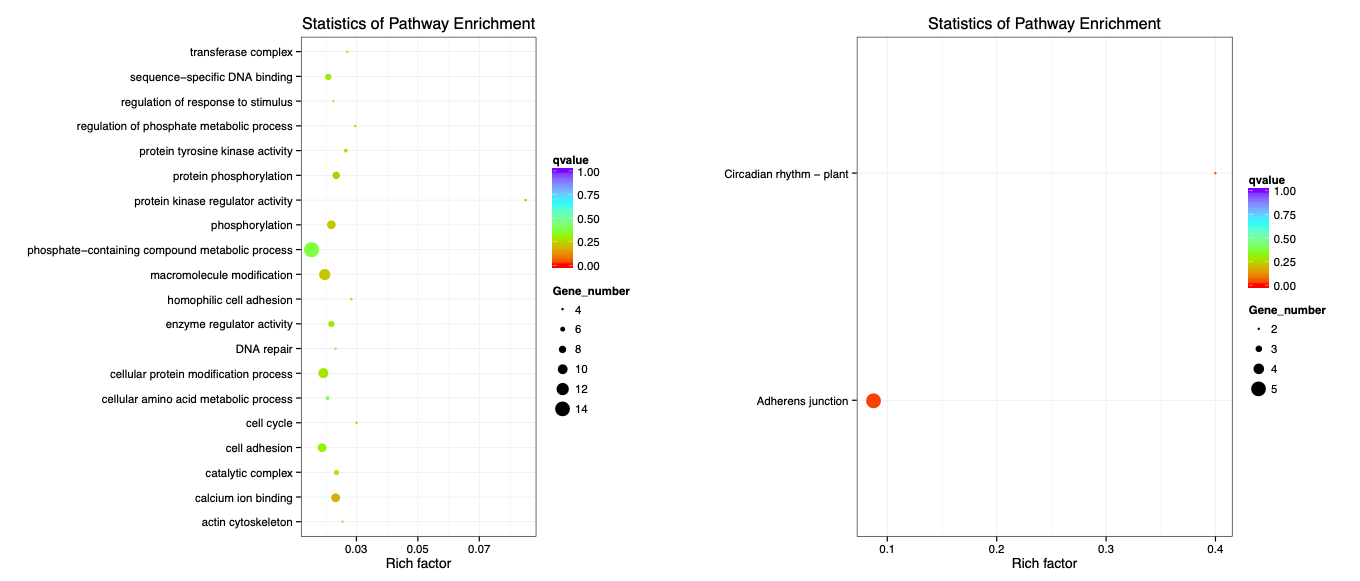
Figure S10 | **Pathway enrichment of positively selected genes for the capability of silk producing**. The foreground branches were *Bmj*, *Bmo* and *Bma* and the background branches were *Aya*, *Har*, *Sfr*, *Pra*, *Dpl* and *Pxy* in the second group . (Left) Results from GO database; (Right) Results from KEGG database.


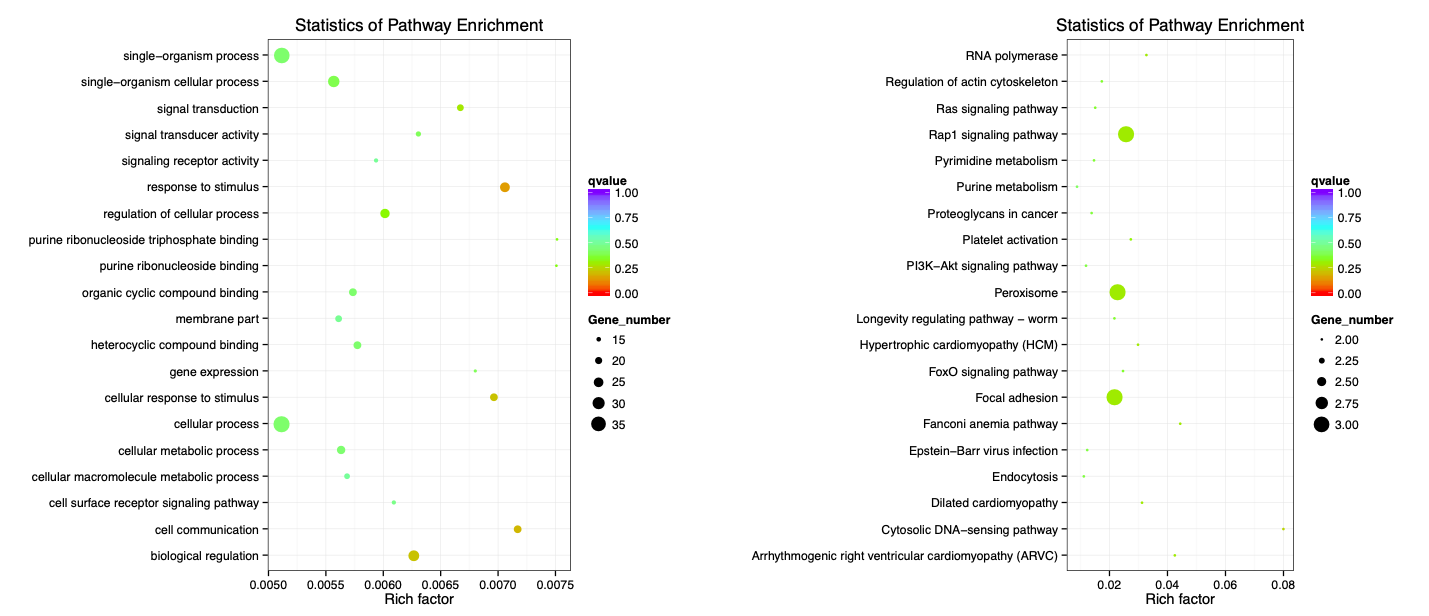


Figure S11 | **Pathway enrichment of positively selected genes** **for the resistance to *BmNPV*.** The foreground branches were *Bmj* while the background branches were *Bmo*, *Har*, *Sfr*, *Pra*, *Dpl*, *Pxy*, *Bma* and *Aya* in the second group . (Left) Results from GO database; (Right) Results from KEGG database.
